# The effect of employment services on employment and health among unemployed persons: an IV approach

**DOI:** 10.1093/eurpub/ckac131.263

**Published:** 2022-10-25

**Authors:** M Schuring, MG Knoef, A Burdorf

**Affiliations:** 1 Erasmus MC, Rotterdam, Netherlands; 2 Leiden University, Leiden, Netherlands

## Abstract

**Background:**

In a large city in the Netherlands, professionals of one department of the municipality focus on rapid return to work, even if that work is not directly in line with education, experience or preference of the client. The first aim was to investigate the effects of this work-first strategy of the municipality on entering paid employment and on health among unemployed persons. The second aim was to determine whether these effects differed between persons, depending on age, sex, education, health, and employment history.

**Methods:**

To identify the effectiveness of employment services of the municipality, a judge leniency instrumental variable (IV) design was used, based on referral behaviour of case workers. In total, 3272 persons who applied for welfare in a large city in the Netherlands in the year 2015, were included in the study. Information on employment services was derived from the municipality. Information on individual characteristics and employment status was derived from register data from Statistics Netherlands. Information on diseases was derived from a medication register. IV regression models and ordinary least squares (OLS) regression models were performed.

**Results:**

The work-first strategy increased the likelihood of entering paid employment by 27% points (b = 0.26, se = 0.07) and had a positive influence on health by 4% points (b = 0.06, se 0.03) of welfare recipients compared to other employment services. Although standard errors were larger for subgroups, the positive effect of the work-first strategy on employment was driven by relatively young (18-30 yrs) and old (45-65 yrs) welfare recipients, with low and intermediate education level.

**Conclusions:**

The instrumental variable approach is a valuable approach to investigate the effect of employment services on entering paid employment and health. To improve health of unemployed persons, it is important to promote entering paid employment.

**Key messages:**

• Active labour market policies, such as the work-first approach, are beneficial for individuals, by increasing employment participation and health, and society, by increasing the employment rate.

• The IV approach is a promising approach to study causal relations in a quasi-experimental study. By using large datasets, differential effects across different groups of participants can be studied.

